# Mixed-Mode Interlaminar Fracture Toughness of Glass and Carbon Fibre Powder Epoxy Composites—For Design of Wind and Tidal Turbine Blades

**DOI:** 10.3390/ma14092103

**Published:** 2021-04-21

**Authors:** Christophe Floreani, Colin Robert, Parvez Alam, Peter Davies, Conchúr M. Ó Brádaigh

**Affiliations:** 1 School of Engineering, Institute for Materials and Processes, The University of Edinburgh, Edinburgh EH9 3FB, UK; colin.robert@ed.ac.uk (C.R.); parvez.alam@ed.ac.uk (P.A.); c.obradaigh@ed.ac.uk (C.M.ÓB.); 2 Ifremer, Marine Structures Laboratory, 29280 Plouzané, France; peter.davies@ifremer.fr

**Keywords:** toughened composites, fracture toughness, delamination

## Abstract

Powder epoxy composites have several advantages for the processing of large composite structures, including low exotherm, viscosity and material cost, as well as the ability to carry out separate melting and curing operations. This work studies the mode I and mixed-mode toughness, as well as the in-plane mechanical properties of unidirectional stitched glass and carbon fibre reinforced powder epoxy composites. The interlaminar fracture toughness is studied in pure mode I by performing Double Cantilever Beam tests and at 25% mode II, 50% mode II and 75% mode II by performing Mixed Mode Bending testing according to the ASTM D5528-13 test standard. The tensile and compressive properties are comparable to that of standard epoxy composites but both the mode I and mixed-mode toughness are shown to be significantly higher than that of other epoxy composites, even when comparing to toughened epoxies. The mixed-mode critical strain energy release rate as a function of the delamination mode ratio is also provided. This paper highlights the potential for powder epoxy composites in the manufacturing of structures where there is a risk of delamination.

## 1. Introduction

Fibre reinforced polymer composites have very good in-plane mechanical properties in the fibre direction. Laminated composites have no through-thickness reinforcement, however, and are therefore subject to the risk of delamination, one of the lowest energy modes of failure [1], making it one of the main causes of concern in composite structures exposed to fatigue loads such as wind and tidal turbine blades. Delamination can also occur in composite structures exposed to high interlaminar stresses. The causes of delamination can be grouped into three main categories [2]: (i) delamination from out-of-plane loading, such as in joints or because of impact loads, (ii) the loading of curved composites which creates out-of-plane stresses and delaminations, and (iii) delamination originating from material discontinuities such as ply drops, holes, or free edges. The study and prediction of delamination in both static and fatigue loading is an active topic of research and has been the subject of numerous publications in recent years [3,4,5]. Preventing delamination is critical to the integrity of a composite structure as its presence drastically reduces its load bearing capabilities [6] and it is difficult to detect [7].

There are three modes of delamination: (i) mode I is the normal crack opening mode, (ii) mode II is in-plane shear delamination along the fibre direction and (iii) mode III is the out-of-plane shear delamination. In most structures, delamination will occur in mixed-mode with combined normal and shear crack propagation. For example, in a wind turbine blade, delamination may occur through a combination of mode II delamination generated by the bending stresses in tension and compression as well as mode I fracture due to the curvature of the deformed blade leading to out-of-plane loading. The rate of delamination in laminated composites is governed by the Strain Energy Release Rate (SERR), defined as the strain energy released per crack surface area as it propagates. It is therefore crucial to select materials with a high toughness for structures which may be exposed to delamination. One option is to use advanced thermoplastic matrices such as Polyetheretherketone (PEEK) or Polyetherketoneketone (PEKK), but due to cost constraints, it is not always economical to produce large structures from these high performance materials. Very large composite structures, such as wind and tidal turbine blades, typically contain numerous ply drops which exacerbate the risk of delamination. Engineering thermoplastic matrices such as Nylon (PA) and Polybutylene terephthalate (PBT) could be considered for these large structures, but their viscosities are typically too high to achieve good consolidation under the vacuum-only processing conditions involved. Recent work in the use of low viscosity, in-situ polymerised thermoplastics such as acrylics have opened the field of manufacture of room-temperature infused large structures from thermoplastic composites [8,9], though there are still fundamental materials and manufacturing issues to be overcome.

The idea of manufacturing composites using powder polymers has been studied since the 1970s [10]. In the following decades, the use of both thermoplastic and thermoset powder resins to produce composite tape [11,12,13] were studied. In recent years, a powder epoxy resin has been developed for the manufacturing of large out-of-autoclave composite structures, focusing initially on the production of wind turbine blades, using oven heating or electrically heated ceramic tooling [14,15]. Recent work has shown that this resin has a low minimal viscosity [16] enabling good fabric impregnation and the manufacturing of structures with low porosity. It also produces a very low exothermic reaction with an enthalpy of 140 to 180 J/g compared to 300 to 500 J/g for conventional epoxy resins [16]. A subsequent study using resin flow and heat transfer models to study degree of cure and temperature profile during the manufacturing of a 100-ply thick laminate showed the potential of this powder epoxy for reducing the thermal runaway caused by the exothermic reaction [17,18]. The processing of powder epoxy composites also produces little to no volatile organic compounds, reducing the potential harm to humans and the environment and it is thermally stable, which means it can be stored at room temperature for long periods of time. Finally, the powder epoxy resin melts around 45 °C while curing only starts around 140 °C [16]. This allows for separation of the melting and curing phases, enabling the individual sections of structures such as turbine blades (e.g., skins and shear webs) to be formed and consolidated separately, assembled and then co-cured in a one-shot process without the need for adhesives [14]. All these properties make powder epoxy a good candidate for the manufacturing of large composite structures. Recent work involving the manufacturing and testing of a 6 m torsion box demonstrator from powder epoxy composites showed that large complex structures can be manufactured with good quality using this resin system [19].

Most of the work related to powder epoxy composites has focused on manufacturing properties and there are fewer data on their mechanical properties. A study on the development of an automated towpregging line using carbon and basalt fibre reinforced powder epoxy showed that the 0° tensile properties of the carbon samples were similar to those published by the fibre manufacturers for standard epoxies [20]. The study also included tension, compression and flexural tests performed at 0° and 90° for the basalt fibre powder epoxy composites which showed that, despite a slightly lower 0° compression strength, the rest of the measured properties were comparable to those of standard epoxies. Another study focused on the influence of fibre straightness and fibre sizing on the mechanical properties of powder epoxy composites [21,22]. This study also included 0° tensile testing as well as 0° and 90° flexural testing. It showed that the powder epoxy reinforced carbon composites performed very well in tension in the longitudinal direction but were found to have a 25% lower 0° flexural strength compared to data published by the fibre manufacturer. This was attributed to a lower longitudinal compression strength than that of standard epoxy composites. The mode I interlaminar fracture toughness was also determined using a Double Cantilever Beam (DCB) test. The values ranged from 1120 J/m${}^{2}$ to 1610 J/m${}^{2}$ at propagation and 950 J/m${}^{2}$ to 1300 J/m${}^{2}$ at initiation, depending on the fibre sizing used. However, no complete study of the mechanical properties of powder epoxy composites manufactured using fabrics has been published. As wind and tidal turbine blades are manufactured using fabric reinforcements, it is essential to obtain their mechanical properties. Additionally, the mixed-mode toughness properties of powder epoxy composites have not been previously investigated.

The aim of this work is to determine the in-plane mechanical properties of unidirectional glass and carbon fibre reinforced powder epoxy composites in tension and compression, as well as their toughness under mode I and mixed-mode loading. This will serve as the basis for the comparison of the mechanical properties of powder epoxy composites with those of standard epoxy composites and assess their potential as a material of choice in areas exposed to high risks of delamination. This work also discusses the differences in the observed behaviour between the carbon and glass composites studied, followed by fractography of the Mixed-Mode Bending (MMB) fracture surfaces using a Scanning Electron Microscope (SEM). Finally, the SERR as a function of mode ratio is measured for both the CFRP and GFRP powder epoxy composites using the Benzeggagh–Kenane and Power Law delamination criteria.

## 2. Experimental Procedure

### 2.1. Material Manufacture and Preparation

Fabrics which are readily available in the market are more likely to be suitable for the manufacturing of wind and tidal turbine blades in the near future than the specialised fabrics often studied in research projects. Fabrics used in this study were therefore limited to typical styles and weights used commercially in wind turbine blade production and were purchased from SAERTEX^®^ (SAERTEX GmbH & Co. KG, Saerbeck, Germany). To avoid issues with unstable crack growth following the weave pattern and hence deviating from single mode delamination characteristics of woven or multi-directional laminates [23], it was decided to work with unidirectional (UD) stitched fabrics, as a first step towards characterisation of the interlaminar fracture toughness properties of fibre reinforced powder epoxy composites. Hence, the following fabrics were studied: (i) a UD stitched carbon fabric (U-C-603 g/m${}^{2}$-1230 mm) with 581 g/m${}^{2}$ Zoltek (ZOLTEK Corporation, Bridgeton, MT, USA) Panex 35-13 50 K 0° fibres, 16 g/m${}^{2}$± 60° E-glass fibres and 6 g/m${}^{2}$ polyester stitching [24]; and (ii) a UD stitched glass fabric (U-E-591 g/m${}^{2}$-1200 mm) with 520 g/m${}^{2}$ E-glass 1.200 Tex fibres, 54 g/m${}^{2}$ 90° E-glass fibres and 17 g/m${}^{2}$ polyester stitching [25].

The formulation of the powder epoxy resin is proprietary and was not made available to the authors. It was designed by Swiss CMT AG and manufactured by Freilacke, Ltd. (Bräunlingen, Baden-Württemberg, Germany) who supplied the resin used in this work.

**Figure 1:**
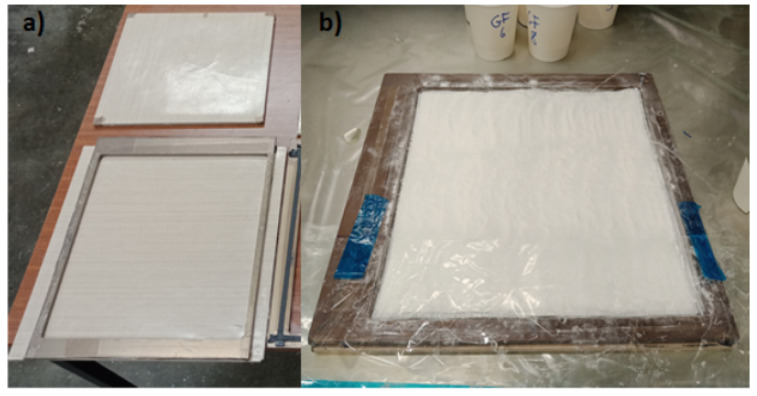
Frame designed for manufacturing fibre reinforced epoxy plates (**a**) before layup (**b**) during DCB plate manufacturing.

As powder epoxy possesses a low minimum viscosity (prior to gelation), it has a tendency to bleed out from the UD composite during the curing stage, which can lead to a high variation in the fibre volume fraction (FVF) of the final parts, making it difficult to manufacture plates at a specific FVF. To avoid this, a frame with inner dimensions of 300 mm × 280 mm was manufactured as shown in Figure 1, from an 8 mm thick stainless-steel plate. Two stainless steel caul plates were used in the process with the top one designed to have a tight fit into the frame, ensuring that uniform pressure was exerted by the applied vacuum, as well as limiting the resin bleed-out. To ensure easy removal of the plates, the caul plates were covered in TFG 250 PTFE (Tygavac Advanced Materials Ltd., Oldham, UK) coated glass fibre and thin TF 050AH PTFE (Tygavac, Oldham, UK) tape was applied to the frame. Powder epoxy was sprinkled evenly before the first ply and subsequently between each ply. The weight of powder epoxy was calculated using the resin and fabric densities to lead to a 50% FVF for the finished plates and was evenly distributed between the plies. This process ensured a good fibre wetting and an even resin distribution. Once the layup was complete, the system was vacuum bagged and cured in an oven. As the powdered resin was stored in the laboratory at room temperature for several months, it was susceptible to humidity absorption. Therefore, the first step of the laminate curing cycle involved drying by exposing the laminate to a 50 °C temperature environment for 8 h. This stage also lowers the viscosity of the resin as it starts to sinter and melt around this temperature allowing for good fibre impregnation. This was followed by 2 h of curing at 135 °C, followed by two hours of post-cure at 185 °C. A dynamic mechanical thermal analysis was conducted on the neat powder epoxy resin on a Tritec 2000 machine produced by Triton Technology (Keyworth, UK). The cured resin was shown to have a Tg onset of 105.5 °C and a Tg defined as the peak of the tan(δ) curve of 125.6 °C.

### 2.2. Tensile Testing

The tensile tests were performed according to BS EN ISO 527 [26] using 0°, 90° and ±45° coupons. The samples were machined using a Benetec^®^ (Barnstaple, UK) diamond coated wet saw to a length of 250 mm and a width of 25 mm. Five coupons were extracted for each test as required by the standard. The test was performed using an MTS Systems Corporation (Eden Prairie, MN, USA) Criterion C45 Universal Test Machine using a crosshead displacement of 2 mm/min and a 300 kN load cell. Glass fibre end tabs were bonded to the specimens to limit stress concentrations at the grips of the MTS machine. Paint was speckled on the coupons to allow for strain measurement with an Imetrum™ (Bristol, UK) Video Extensometer.

The stiffness and Poisson’s ratio were determined over a strain range of 0.05% to 0.25%. The shear modulus was calculated from the ±45° samples using Equation (1) [27]:

(1) $$G_{12}=\frac{E_{x}}{2(1+\nu_{xy})}$$

where: $G_{12}$ is the material shear modulus, $E_{x}$ is the stiffness in the global x-direction corresponding to the 0° direction in this case and $\nu_{xy}$ is the Poisson’s ratio in the global coordinates.

### 2.3. Compression Testing

**Figure 2:**
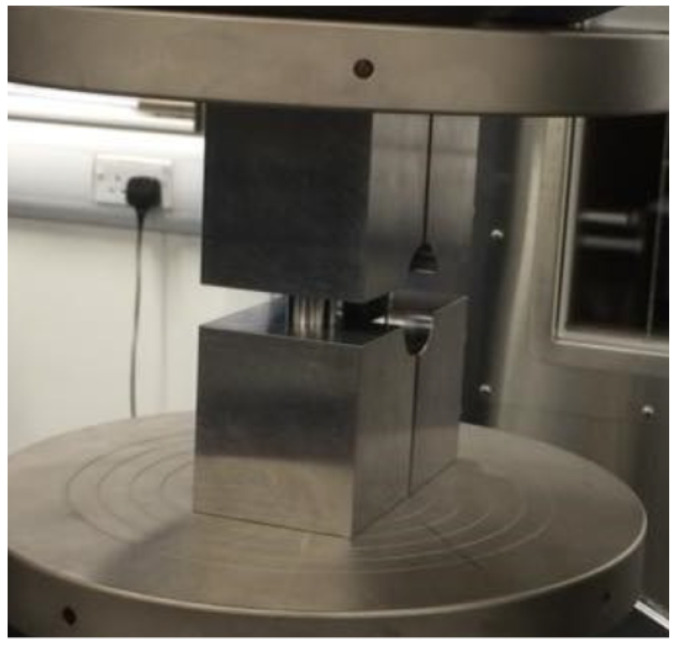
Compression Testing According to ASTM D6641.

Compression testing was carried out according to ASTM D6641 [28] which allows the determination of the compressive strength and stiffness of fibre reinforced composites using a Combined Loading Compression (CLC) fixture. Five 0° and 90° samples with a width of 13 mm and length of 140 mm were extracted from the four-ply CFRP and six-ply thick GFRP plates. The coupons were tested untabbed using a CLC fixture manufactured by Wyoming Test Fixtures (Salt Lake City, UT, USA) shown in Figure 2, an MTS Criterion C45 Universal Test Machine with a crosshead displacement of 1.3 mm/min and a 300 kN load cell. Paint was also speckled on the compression samples to allow for strain measurement using the Imetrum™ video extensometer.

### 2.4. Mode I Interlaminar Fracture Toughness

**Figure 3:**
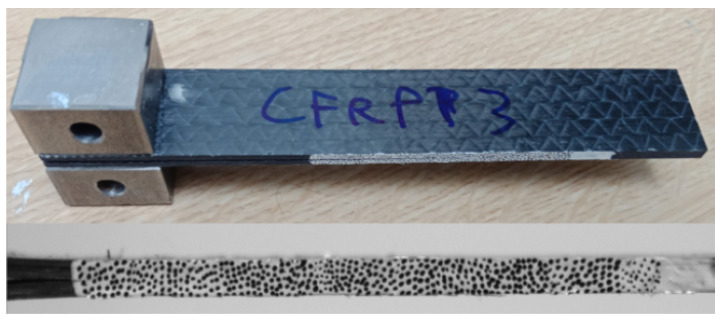
Specimen manufactured for double cantilever beam testing.

A Double Cantilever Beam (DCB) test was conducted on the CFRP samples following ASTM D5528-13 [29]. This allowed the measurement of the critical mode I strain energy release rate during crack initiation ($G_{ic}$) and propagation ($G_{IC}$). The test was conducted on a 3369 series Instron^®^ (High Wycombe, UK) 50 kN Universal Test Machine equipped with a 1 kN load cell. Stainless steel loading blocks were bonded to the ends of the composite using VTFA 400 adhesive and a 1 mm/min constant crosshead displacement was applied. The CFRP plates were manufactured with six plies giving an average specimen thickness of 3.52 mm, while the GFRP plates were manufactured with eight plies for an average specimen thickness of 3.47 mm. A 60 mm long, 13-μm thick Teflon (PTFE) film was inserted in the middle of the plate during manufacture as shown in Figure 1b). White paint was applied to the side of the specimen as seen in Figure 3, on which a random pattern of black dots was added. This allowed the Manta G-146B/G-146C camera (Allied Vision Technologies GmbH, Stadtroda, Germany) and the Imetrum video extensometer to record the crack extension in real time. Considering that the loading blocks were 25 mm in length, the DCB samples had an initial crack length around 47.5 mm. The initial crack length was measured at the end of each test to obtain the precise value needed for the $G_{IC}$ calculations.

Using the Modified Beam Theory (MBT) method, the $G_{IC}$ can be calculated given by the following equation [29]:

(2) $$G_{IC}=\frac{3P\delta }{2b(a+|\Delta |)}.\frac{F}{N}$$

where *P* and δ are respectively the load applied and displacement at the loading blocks, *b* is the specimen width, *a* is the crack length and |Δ| is a correction factor which can be determined by plotting the linear best fit of the cube root of the compliance against the crack length and calculating the root of this function. The MBT method uses this correction factor to artificially increase the delamination length used in the $G_{IC}$ calculation. It accounts for the rotation of the crack front. *F* is a large displacement correction factor and *N* is a loading block correction factor. Equations to calculate *F* and *N* are defined in the ASTM D5528-13 [29] test standard.

Results for critical strain energy release rate ($G_{IC}$) are reported both at the point of fracture initiation and crack propagation as recommended in the ASTM D5528-13 test standard. Three different definitions were proposed for the point of fracture initiation from the starter film (with no pre-crack): (i) the location in the load-displacement curve where there was a deviation from linearity (referred to as NL in this paper); (ii) the point at which delamination growth was observed visually; (iii) the point at which the compliance of the specimen increased by 5% (referred to as 5% compliance in this paper). In this work, the initiation $G_{IC}$ values were reported using the NL point and the 5% compliance point. The propagation $G_{IC}$ was defined as the mean of the R-curve values from the 5% compliance point until the end of the test.

### 2.5. Mixed Mode Interlaminar Fracture Toughness

**Figure 4:**
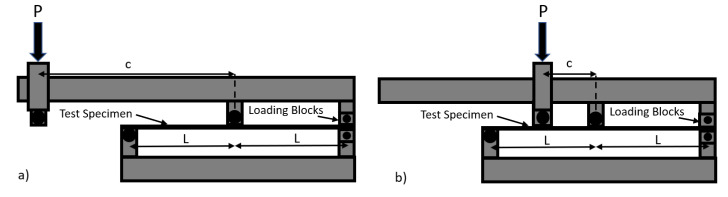
MMB test fixture showing (**a**) 25% Mode II and (**b**) 75% Mode II.

Mixed-Mode Bending (MMB) tests were carried out according to ASTM D6671 [30]. To obtain enough points to obtain a critical strain energy release rate curve as a function of the mode ratio (ϕ), defined as the ratio of mode II strain energy release rate over the total SERR, it was decided to perform three sets of tests: 25% mode II, 50% mode II and 75% mode II. These three ratios were selected to ensure that the critical SERR was measured at evenly spaced points between 0% and 75% mode ratio. This will allow for good curve fitting of the critical SERR as a function of the mode ratio. The mode ratios (ϕ) were adjusted by adjusting the distance between the load application and the centre of the specimen span as shown Figure 4. In Figure 4a) the load is applied close to the centre of the 120 mm span which represents the 25% mode II while in Figure 4b) the load is applied much further from the centre of the span and was captured from the 75% mode II test. The further the load was applied from the centre of the MMB sample, the higher the percentage of mode I delamination was applied.

The distance from the point of load application to the centre of the sample span(c) is defined in the ASTM D6671 [30] test standard as:

(3) $$c=\frac{12\beta^{2}+3\alpha +8\beta \sqrt{3\alpha }}{36\beta^{2}-3\alpha }$$

where:

(4) $$\alpha =\frac{1-\phi }{\phi }$$

(5) $$\beta =\frac{a+\chi h}{a+0.42\chi h}$$

where *a* is the initial crack length, ϕ is the mode ratio and χ is the crack length correction parameter defined as [30]:

(6) $$\chi =\sqrt{\frac{E_{11}}{11G_{13}}\left(3-2{\left(\frac{\Gamma }{1+\Gamma }\right)}^{2}\right)}$$

where $E_{11}$$G_{13}$ are the longitudinal stiffness and out of plane shear modulus of the sample and Γ is the transverse modulus correction parameter defined as [30]:

(7) $$\Gamma =\frac{1.18\sqrt{E_{11}E_{22}}}{G_{13}}$$

**Figure 5:**
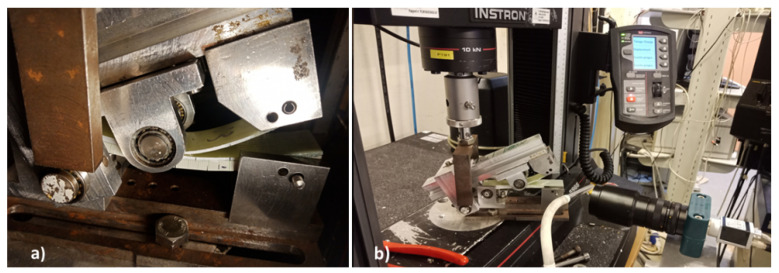
MMB test fixture. (**a**) Closeup of test fixture (**b**) Full MMB setup with instron test machine and camera.

Five samples were extracted from the CFRP and GFRP plates for each test. To maximise the length of crack propagation, the samples were cut to a length of 180 mm so that a 120 mm span length could be used for the MMB test. The same stainless steel loading blocks used during the DCB tests were bonded on the MMB samples. Considering the initial crack length of 47.5 mm this ensured that each sample had around 12.5 mm of crack propagation. This allowed the SERR to stabilise during the test so that both initiation and propagation $G_{c}$ values could be measured. The geometry of the MMB test fixture made it difficult to use a video extensometer to track the crack propagation. Therefore, graph paper was instead glued at the bottom of each sample and a picture was captured each second with a camera to allow for manual measurement of crack length throughout the test. The load and crosshead displacements were measured using a 3369 series Instron^®^ (High Wycombe, Buckinghamshire, UK) 50 kN Universal Test Machine, equipped with a 10 kN load cell. A crosshead displacement of 2 mm/min was applied to the samples. The test setup is shown in Figure 5. The SERR can be calculated throughout the test using the following equations [30]:

(8) $$G_{I}=\frac{12P^{2}{\left(3c-L\right)}^{2}}{16b^{2}h^{3}L^{2}E_{1f}}\left(a+\chi h\right)$$

(9) $$G_{II}=\frac{9P^{2}{\left(c+L\right)}^{2}}{16b^{2}h^{3}L^{2}E_{1f}}\left(a+0.42\chi h\right)$$

where *P* is the applied load, *c* is the distance between the point of load application and the centre of the specimen span, *L* is the half-span of the specimen, *a* is the measured crack length, *h* is the sample half-thickness, *b* is the sample width and $E_{1f}$ is the flexural stiffness in the longitudinal direction estimated from the linear region of the MMB force-displacement curves. The method for obtaining an estimate of $E_{1f}$ is described in the ASTM D6671 test standard [30]. For the MMB test, the reported $G_{c}$ values were defined in a similar way to the DCB test with the initiation $G_{c}$ defined at both the point of non-linearity (NL) and at the 5% increase in compliance point. The propagation $G_{c}$ values were also defined as the mean of the R-curve from the 5% increase in compliance point until the end of the test. However, the shape of the samples used in the MMB test allowed for a stable crack propagation of only 12.5 mm. Therefore, to perform a fair comparison between the mode I and mixed-mode critical strain energy release rates in Section 4.3, propagation values from the mode I test were limited to data from the first 12.5 mm of crack growth only. Finally, using the DCB and MMB test results, the SERR was plotted as a function of the mode ratio for the 0%, 25%, 50% and 75% mode II. The aim of this test is to perform a curve fitting to obtain an estimate of the critical SERR for any mode ratio, as required for a mixed mode delamination finite element model. The data points were fitted using two widely used methods for estimating the mixed-mode SERR: (i) the Power Law criterion [31] and (ii) the Benzeggagh–Kenane (B-K) equation [32]. When mode III delamination can be neglected, the Power Law is defined as [31]:

(10) $${\left(\frac{G_{I}}{G_{IC}}\right)}^{\alpha }+{\left(\frac{G_{II}}{G_{IIC}}\right)}^{\alpha }=1$$

The critical strain energy release rate can be found using the following equation:

(11) $${\left[{\left(\frac{1-\phi }{G_{IC}}\right)}^{\alpha }+{\left(\frac{\phi }{G_{IIC}}\right)}^{\alpha }\right]}^{-1/\alpha }=G_{c}$$

where ϕ is the mode ratio defined as the ratio of mode II SERR divided by the total SERR.

The Benzeggagh–Kenane equation is defined as follows [32]:

(12) $$G_{IC}+\left(G_{IIC}-G_{IC}\right)\phi^{n}=G_{c}$$

where $G_{IC}$ and $G_{IIC}$ are defined as the critical mode I and mode II SERR respectively and ϕ is the mode ratio.

### 2.6. Manufacturing Quality Check

The thickness of each plate tested was measured at 16 different points using a Kroeplin^®^ (Schlüchtern, Germany) thickness gauge to investigate the presence of defects or resin rich regions. The six-ply thick CFRP and eight-ply thick GFRP plates had an average thickness of 3.52 mm and 3.47 mm respectively, with measured standard deviations of 0.077 mm and 0.014 mm respectively. This suggests that the manufacturing process developed produced laminates of consistent thickness with only minor disparities within each plate.

A resin burn-off test according to ASTM D3171-15 [33] was performed to measure the Fibre Volume Fraction (FVF) of the CFRP and GFRP specimens. FVF measurements were taken for a total of six CFRP and GFRP samples. Despite an initial target of 50% FVF, resin bleedout during manufacturing led to average measured FVF values for the CFRP and GFRP samples of 53.3% and 51.1% respectively. There was slightly more resin bleedout for the CFRP plates compared to the GFRP plates, which explains the higher FVF obtained.

**Figure 6:**
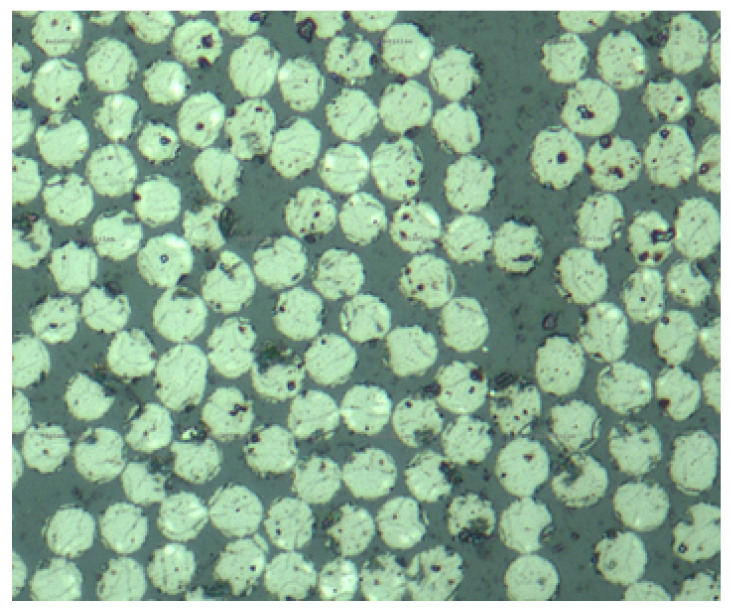
Optical microscope imageof CFRP sample at 50× zoom.

Small 20 mm by 15 mm samples were cut in the transverse fibre direction. They were placed within an epoxy resin matrix, which was cured at room temperature for 24 h, followed by 5 h of post-cure at 50 C. The samples were then polished on an automatic polishing machine using sandpaper with increasing grit size (P400, P800 and P1200). To ensure a very smooth surface for optical micrographs, the samples were polished using a diamond-based dispersion with a 3 μm particle size. The laminates were then observed under a Zeiss optical microscope fitted with an AxioCam™ MRc 5 camera (Oberkochen, Germany). The fibres and resin were observed up to 50× zoom as shown in Figure 6. The black marks around some of the fibres are machining marks which were not successfully removed by the polishing process. However, observations show that there were no visible macro voids in the samples studied. Indeed, these would appear as dark circular spots on Figure 6. Although a more precise imaging method such as a CT-scan would be required to confirm the exact void content, the results from the optical micrographs suggest that the void content is very low for these laminates.

## 3. Results

### 3.1. Tension Results

Table 1 summarises the tensile test results for the carbon fibre and glass fibre powder epoxy specimens. The 0° CFRP coupons showed relatively high variability with a coefficient of variation (COV) of 13.7%. Inspection of the failed samples showed that there were slight deviations in the fibre alignment within the CFRP plates. The use of a heavy 581 g/m${}^{2}$ fabric and the presence of only 3% weight for the off-axis fibres and stitching meant the fibres were very loose during manufacturing, leading to occasional distortions in orientation prior to curing. On the other hand, the glass fibre tensile tests showed low coefficient of variations for all tests (all below 8.4%) suggesting very good repeatability between the coupons. The presence of the 10% of 90° fibres in the glass fabric explained the higher measured stiffness and strength in the transverse direction compared to the CFRP specimens.

### 3.2. Compression Results

The results of the compression tests are summarised in Table 2. The glass fibre coupons showed reductions of 4.1% and 2.2% respectively for the longitudinal and transverse stiffness between the tension and compression tests. The reduction in stiffness between that measured in tension and in compression was far greater for the CFRP samples which showed drops of 18% and 20% respectively for the longitudinal and transverse stiffness. A notable reduction in the longitudinal strength was measured in compression compared to tension with a 48% and 59% drop respectively for the GFRP and CFRP samples. On the other hand, an increase of 70% and 313% respectively was measured for the GFRP and CFRP transverse strength in compression compared to tension. The presence of the off-axis fibres in the GFRP fabric explain the lower increase in the transverse strength in compression compared to the CFRP samples as their tensile transverse strength was more than three times higher than that of CFRP. The variability between samples in the longitudinal direction during compression testing was slightly lower for the GFRP specimens compared to the CFRP, but the difference was much lower than in the tension tests.

### 3.3. Mode I Interlaminar Fracture Toughness

The Double Cantilever Beam (DCB) test results were processed according to ASTM D6671 using the Modified Beam Theory Method (MBT) given in Equation (2). The results are summarised in Table 3 where STD represents the standard deviation and COV the coefficient of variation. By coupling the video extensometer data to the test machine, it was possible to obtain the Force, Displacement and Crack Lengths in real time with a frequency of 0.1 Hz. The DCB tests allowed between 40 mm and 60 mm of crack propagation so the propagation data were averaged over a large range of crack growth. The propagation $G_{IC}$ was measured as 1684 ± 71 J/m${}^{2}$ and 2067 ± 285 J/m${}^{2}$ respectively for the CFRP and GFRP samples. The critical initiation SERRs were measured as 1377 ± 103 J/m${}^{2}$ and 1643 ± 112 J/m${}^{2}$ for the GFRP samples using the NL method and 5% compliance method. Using these same methods, the critical initiation SERRs for the CFRP samples were measured as 851 ± 74 J/m${}^{2}$ and 1070 J/m${}^{2}$. The $G_{IC}$ was therefore higher for the GFRP samples compared to the CFRP for both the initiation and propagation values. The NL, 5% compliance and propagation $G_{IC}$ were 62%, 54% and 22% higher respectively for the GFRP samples.

**Figure 7:**
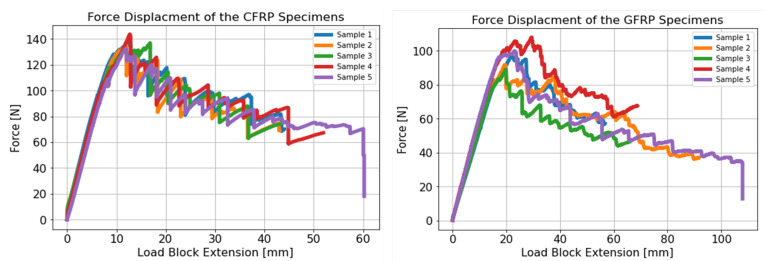
Load-Displacement curves for the DCB samples.

**Figure 8:**
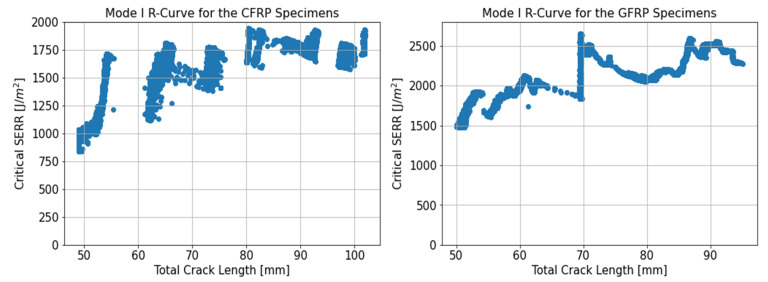
Representative Mode I R-curves for CFRP and GFRP specimens.

The force-displacement curves obtained at the loading blocks are shown in Figure 7. There was variability in the load-displacement curve throughout the test, highlighting the need to use as large a crack propagation as possible to obtain an accurate estimate of the propagation $G_{IC}$. The CFRP samples all had force-displacement plots which were increasing linearly until about 110 N followed by a non-linear increase to a peak around 135 N after which a sudden drop in the load of around 20 N occurred. During the remainder of the test, the force at the loading blocks went through successive periods of steady increase followed by rapid drops. This characteristic stick-slip behaviour is visible in the R-curves shown in Figure 8, in which the SERR was seen to fluctuate between linear increases followed by sudden reductions. Similar R-curves were noted for various resin materials such as PEEK [34], or epoxies [35]. The R-curves showed an increasing trend in the SERR in the first 20 to 30 mm of crack growth for the CFRP samples followed by area period over which the SERR seemed to have stabilised, despite the peaks and troughs in the SERR data. The GFRP samples showed more variability in the force-displacement curves with the peak load varying from 90 N to 105 N. The subsequent reduction in the load was much smoother than the CFRP samples. However, in some of the samples, large increases in the load were visible in the crack propagation region. In the example R-curve shown in Figure 7 for the GFRP samples, this increase in load was visible in the form of a rapid, near vertical increase in the SERR. The trend in the GFRP R-curves was less clear than for the CFRP samples. There was an initial increase in the SERR in the first 5 to 10 mm of crack growth. However, following this initial crack extension, the R-curves showed varying trends with some showing a plateau, while others had sudden increases followed by reductions in the measured SERR.

### 3.4. Mixed-Mode Interlaminar Fracture Toughness

**Figure 9:**
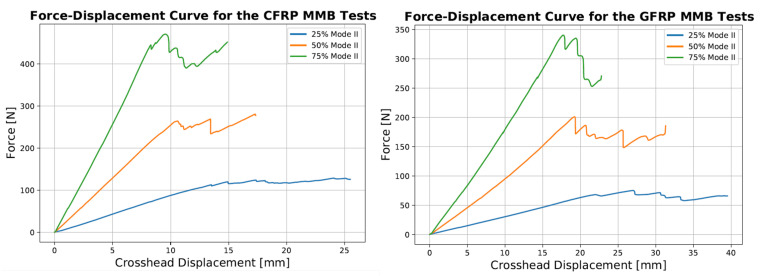
Representative Force-Displacement Curves for the GFRP and CFRP MMB tests at the different mode ratios.

Prior to the start of the test, the appropriate lever arm lengths had to be selected to ensure the experiment was performed at the desired mode ratio. Using Equations (3)–(7), the arm lengths were determined and are summarised in Table 4. The MMB test standard [30] recommended using the value of $G_{12}$ instead of $G_{13}$ if the out-of-plane shear modulus was not available, provided the studied materials were unidirectional. For the GFRP samples, values of $E_{1}$ = 39.4 GPa, $E_{2}$ = 13.7 GPa and $G_{12}$ = 3.91 GPa were used for Equations (6) and (7) while values of $E_{1}$ = 123.0 GPa, $E_{w}$ = 8.47 GPa and $G_{12}$ = 4.30 GPa were selected for the CFRP samples. These values were obtained during tensile testing performed prior to the MMB tests and highlight the importance of knowing the in-plane material elastic properties to obtain an accurate estimate of the mode ratio. Figure 9 shows an example of the Force-Displacement curves obtained at the three mode ratios for the CFRP and GFRP samples. The load at delamination onset and propagation increased with mode ratio while the displacement decreased. Using Equations (8) and (9), the mode I, mode II and total SERR were measured at the point of Non-linearity (NL), the 5% compliance point and at every subsequent point throughout the test until the crack length reached 60 mm. Above this value, the crack propagated beyond the point of contact between the roller at the mid-span of the sample so it was no longer a delamination test, with the upper arm of the sample being loaded in bending. The MMB test results are summarised in Table 5 and Table 6.

**Figure 10:**
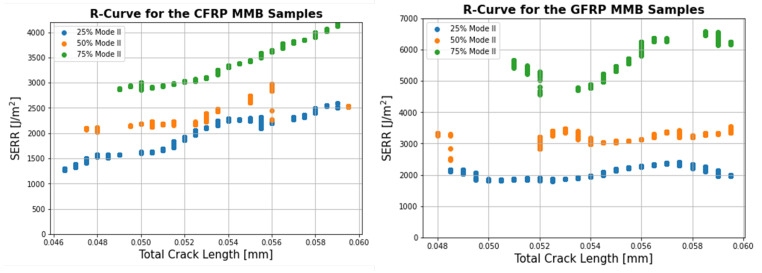
Representative R-Curves for the CFRP and GFRP samples at the 3 Mode Ratios.

Example R-curves from the point of 5% compliance until the crack length reach 60 mm are shown in Figure 10. For this range of crack extension, the R-curves for the three different mode ratios for CFRP displayed an increasing trend from the 5% compliance point until the end of the test. The R-Curves for the GFRP samples were flatter, despite some fluctuations and showed no clear trends despite some notable differences between the samples. The shorter distance of crack propagation of between 10 and 15 mm, depending on the samples, results in fewer values over which to average the SERR. Therefore, care needs to be taken comparing the mean R-curve results of the DCB test to the mixed-mode test. This is why comparison between mode I and mixed-mode SERR data will be carried out using the initiation $G_{IC}$ as well as a mean R-curve value from the DCB tests for the first 12.5 mm of crack growth.

## 4. Discussion

### 4.1. Performance of Powder Epoxy Compared to Standard Epoxy in Tension and Compression

The performance of the powder epoxy carbon fibre samples can be evaluated by using a material datasheet from Zoltek™ [36] which lists the longitudinal mechanical properties of UD Zoltek PX35 unidirectional fabrics manufactured using standard epoxy resin with an FVF of 55%. This was used to compare the properties obtained using standard epoxy resin. As the datasheet did not specify the exact composition of the resin, fabric, and manufacturing method, it was difficult to perform a rigorous comparison between the two epoxy systems. However, the obtained longitudinal strength and stiffness of 1492 MPa and 123 GPa respectively measured here were higher than the 1400 MPa and 119 GPa listed by the manufacturer, suggesting that despite slight distortions in the fibre alignment within the samples, a good manufacturing quality was achieved for the samples, despite a slightly lower FVF (51.1% vs. 55% in the data sheet). Although it is difficult to find mechanical properties in literature for glass fibre reinforced epoxies with the same fibre architecture, the powder epoxy reinforced glass fibre composites performed well compared to conventional epoxy systems [37,38].

The reductions in longitudinal and transverse stiffness observed in compression testing of the CFRP samples of 18% and 20% respectively were more than expected [36] and are perhaps linked to the slight distortion of the fibre orientation during manufacturing. During a tensile test, these fibres are straightened in the initial part of the test, leading to a higher stiffness while this phenomenon does not occur in a compression test. A 59% reduction in the longitudinal strength was measured in compression compared to tension as compared to the 30% reduction reported in the Zoltek datasheet with a compressive strength of 980 MPa [36]. Another study performed on pultruded Zoltek PX35 with epoxy resin showed a reduction in the compressive strength of 43% to a value of 897 MPa [39]. On the other hand, the glass fibre samples showed only a minor reduction in the longitudinal stiffness in compression, suggests that the measured lower stiffness of the CFRP in compression is not related to poor performance of the powder epoxy. The measured compressive strength was 48% lower than the tensile strength. In a study on the influence of fibre volume fraction (FVF) on the tensile and compressive properties of glass/epoxy composites [40], the compressive strength at 50% FVF was reported as 600 MPa compared to 900 MPa for the tensile strength, representing a 33% difference. In another study [41], representative mechanical properties for E-glass epoxy with 60% FVF show a tensile and compressive longitudinal strengths of 1020 MPa and 600 MPa for a reduction of 39%. The glass fibre/powder epoxy composite had a compressive strength that was 14% lower than these two materials.

Therefore, the tensile and compression test results for both the glass and carbon reinforced powder epoxy composites show that the in-plane mechanical performance of these composites is comparable to and, in some cases, better than that of conventional epoxy composites despite a slightly lower compressive longitudinal strength.

### 4.2. Differences in Measured Toughness between GFRP and CFRP Specimen

The aim of this work is to characterise the mixed-mode fracture toughness properties of powder epoxy composites reinforced with carbon and glass fibres. It is not possible to perform a direct rigorous comparison between the performance of the CFRP and GFRP samples, however, as mentioned above, because of differences in the reinforcing fabric architecture. Nevertheless, the differences in behaviour can be discussed as well as possible explanations for these observed differences.

**Figure 11:**
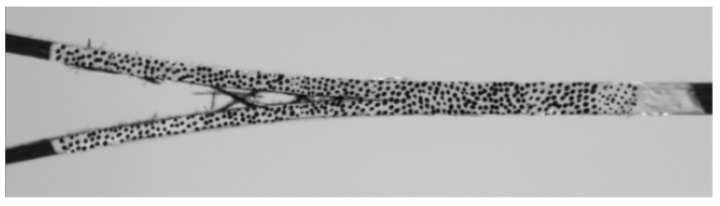
CFRP DCB sample during testing with off-axis fibre bridging.

**Figure 12:**
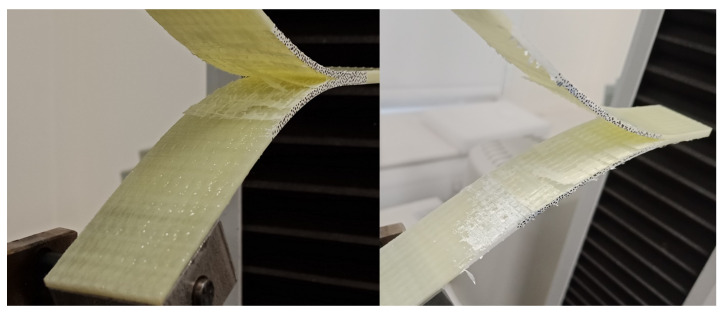
GFRP sample during testing with 90° fibre bridging.

The carbon and glass fibre fabrics used in this study have different configurations, even though both are classified as unidirectional (UD). In most studies on the interlaminar fracture toughness properties found in literature, the precise composition of the UD fabrics is not specified, despite most having off-axis fibres and stitching to keep the fibres in the correct alignment during manufacturing. The UD carbon fabric tested here has ±60° degree fibres and stitching representing 2.6% and 1.0% of the areal weight respectively while the UD glass fabric has 9.1% content of 90° fibres, as well as 2.9% stitching. The off-axis fibres in the carbon fabric clearly play a role, most visibly in the DCB test as evidenced in Figure 11. The presence of these fibres may explain the observed stick-slip behaviour on the force-displacement curves and the corresponding peaks and troughs of the R-curves. As the crack propagates, the ±60° fibres are pulled out and bridge the crack, therefore artificially delaying propagation and increasing the apparent SERR. This is followed by breakage of these fibres, leading to sudden drops in both the measured force. In the GFRP specimens, as the off-axis fibres were at a 90° angle, the bridging mechanism is different. Fibre bridging is no longer visible from the side view of the sample as captured by the video extensometer, but as shown in Figure 12, they still impede crack growth and, in some cases, result in a non-uniform crack front. Figure 12 also shows that the presence of the 90° fibres sometimes forces damage to spread from the interlaminar region into the adjacent ply, which could explain the sudden increases in the measured SERR observed in the R-curve shown in Figure 8 where crack propagation was momentarily arrested. This may also explain the higher variability observed in the GFRP samples with a COV of 13.1% compared to 4.2% for CFRP specimens, as the fibre bridging is less uniform during crack propagation.

The glass fibre samples showed higher initiation and propagation values for the mode I and the three different mode ratios studied in this work, except for the mean R-curve value for the 25% mode II test. A possible explanation may be the high standard deviation measured in these samples, or perhaps that the R-curve takes longer to increase to a stable value in the GFRP samples in a test where only the first 13 mm of crack growth were observed. The largest absolute difference in the measured critical SERR, however, was for the 75% mode II case. This is probably explained by the presence of the 90° fibres in the GFRP samples impeding crack propagation in mode II dominated failure more effectively than the ±60° fibres found in the CFRP samples. The difference in orientation of the off-axis fibres as well as the higher percentage of these fibres in the UD GFRP fabric is believed to be the main factor in the higher measured SERR both at initiation and propagation. Other factors, however, such as the difference in stiffness between carbon and glass fibres, cannot be excluded. In this work, CFRP and GFRP samples were studied primarily with the aim of being able to compare their relative performances under mixed-mode loading with regards to other GFRP and CFRP composites results found in the literature rather than with each other.

### 4.3. Fractography of MMB Samples

**Figure 13:**
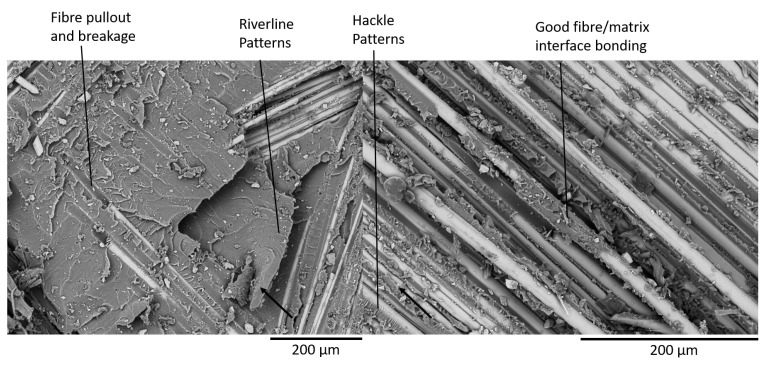
25% Mode Ratio Fracture Surface of GFRP Specimen (Crack Direction shown by the Arrow).

**Figure 14:**
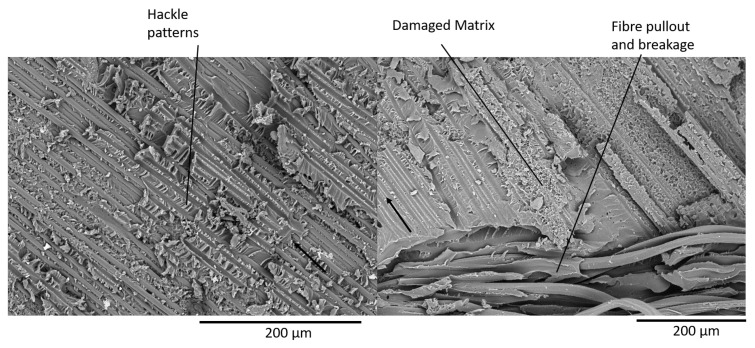
75% Mode Ratio Fracture Surface of CFRP specimen (crack direction shown by the arrow).

After testing, the MMB samples were coated in a thin layer of gold and observed under a TM4000Plus Scanning Electron Microscope (SEM) manufactured by Hitachi (Tokyo, Japan). The most notable difference in the fracture surfaces of the glass and carbon powder epoxy composites is the presence of a greater number of pulled-out fibres in the glass specimens. This is caused by the higher number of off-axis fibres in the glass fabric. However, most of the failure phenomena observed were similar for both glass and carbon samples. Therefore, SEM pictures of the 25% mode ratio for the GFRP samples (Figure 13), and the CFRP 75% mode ratio (Figure 14) were used to illustrate the main fracture mechanisms which occurred during delamination.

Hackle patterns, characteristic of mode I delamination are found on both the GFRP and CFRP fracture surfaces as shown in Figure 13. These features were also observed in literature for mode I dominated delamination tests [42,43]. Hackle patterns, characteristic of mode II delamination [32,43] are also visible in the 25% mode ratio specimens but are far more present in the 75% mode ratio samples as shown in Figure 14. Fibre pull-out and breakage, which were visible in Figure 11 and Figure 12 are present on the fracture surfaces of both the 25% and 75% mode ratio samples.

Another interesting feature observed during SEM analysis is the presence of a highly damaged matrix. This was slightly more prevalent in the 75% mode ratio samples but it was also found in the samples tested at 25% mode ratio. This feature, present in both the GFRP and CFRP samples, has not been reported in the literature. It may perhaps explain the very high toughness measured in powder epoxy composites. Additionally, all samples displayed a fracture surface with a high roughness, suggesting that the crack progression was not smooth along the length of the sample.

### 4.4. Comparison of Powder Epoxy SERR with Other Resin Systems

A list of mixed-mode critical initiation SERR values found in a literature survey is summarised in Table 7. As the different studies were performed at various mode ratios, the relevant mode ratios for each material are specified. In most cases, the reported SERR corresponds to the initiation SERR, although it is not always clear if that corresponds to the NL point or the 5% compliance point. Table 7 does not aim to present an exhaustive list of results, but rather to show representative results obtained for the mixed-mode toughness of both standard and toughened epoxy composites. As mentioned in Section 4.1, in most cases, the exact fabric fibre architecture of the studied materials is unknown and therefore direct comparisons are not possible. It is clear from the presented results, however, that powder epoxy composites perform very well in terms of SERR, even when compared to epoxies toughened with rubber or even multi-walled carbon nanotubes. Indeed, the mode I SERR measured at the NL point was more than double for the CFRP and GFRP powder epoxy composites compared to the reported values in Table 7. This is also true for the mixed-mode tests, with the CFRP and GFRP powder epoxy showing critical Gc values of up to three times those reported in the literature even for toughened epoxy composites. This study therefore shows that powder epoxy composites have toughness that is higher than most toughened epoxies available on the market. To the knowledge of the authors, no published results showed an epoxy composite with higher toughness than these powder epoxy composites. It is therefore a very good material choice in structures exposed to a high risk of delamination.

### 4.5. Mixed Mode Bending Criteria

**Figure 15:**
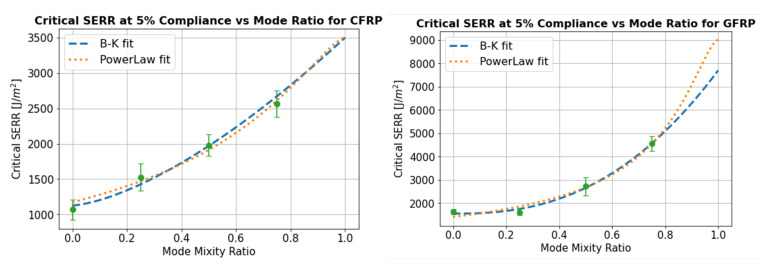
Initiation: SERR at 5% compliance vs. mode ratio for the CFRP and GFRP samples.

**Figure 16:**
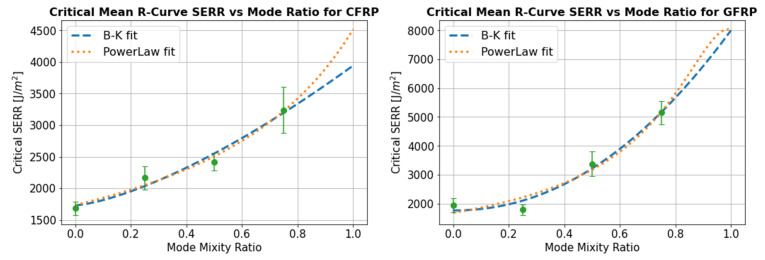
Propagation: mean R-curve SERR vs. mode ratio for the CFRP and GFRP samples.

The Power Law and B-K curve fitting of the critical SERR vs mode ratio are shown in Figure 15 for the 5% compliance initiation point and in Figure 16 for the propagation SERR defined as the mean R-curve from the point of 5% compliance until the crack has propagated to a total length of 60 mm. The exponent *n* in the B-K curve fit was found to be equal to 1.49 and 1.43 in the CFRP samples at the 5% compliance and propagation SERR exponent in the Power Law, α, was found to be equal to 1.19 and 0.97. For the GFRP specimens, the BK exponent was equal to 2.47 and 2.10 for the 5% compliance and propagation SERR values respectively while the Power Law exponent had a value of 1.39 and 1.54.

Using either of these fitting methods, it is possible to estimate the critical SERR value for any mode ratio. This may therefore serve as the input to a Finite Element delamination analysis, and these two curve fitting methods were selected because they are incorporated in some of the widely used commercial FE packages such as Abaqus™ and Ansys™. It is difficult to comment on the relative performance of the B-K and Power Law curve fitting methods as there is data presented for only four different mode ratios. Additionally, in the absence of 100% mode II data, the mode II SERR was introduced as a bounded variable during curve fitting. In any case, the existence of pure mode II test values is open to discussion, due to the influence of friction [51] so it may be more reliable to base estimations for $G_{IIC}$ on mixed mode extrapolations. Generally, mode II SERR was estimated to be higher using the Power Law compared to the B-K fit. However, apart from the mode ratios close to 1, the estimated SERR were very similar with only minor differences between the two approaches. The variability in the SERR experimental data is much higher than the differences obtained between the two curve fitting methods, suggesting that both would be acceptable as an input to a mixed mode delamination analysis.

## 5. Conclusions

Powder epoxy has been shown in previous work to have very good properties for the manufacturing of large composite structures [14,15], with low minimal viscosity [16,17,18], low exotherm [16], no VOCs released, the capacity for long term room temperature storage and its suitability for out-of-autoclave manufacturing. As part of this study, unidirectional stitched carbon fibre and glass fibre composites reinforced with powder epoxy were manufactured. The quality of the specimens was found to be adequate for a study on the mechanical properties, with no observable voids, consistent thicknesses, and low variations of the fibre volume fractions between the samples.

The tensile and compressive in-plane mechanical properties of the powder epoxy composites were determined for these samples and are shown to be comparable to those of commonly used epoxy resin systems. The interlaminar fracture toughness was studied in pure mode I by performing Double Cantilever Beam tests and at 25% mode II, 50% mode II and 75% mode II by performing Mixed Mode Bending testing. The SERRs at both crack initiation and propagation were shown to be significantly higher than both conventional and toughened epoxy composites for which published data is available, with initiation $G_{C}$ values ranging between 1377 J/m${}^{2}$ and 3118 J/m${}^{2}$ for the GFRP powder epoxy composites and from 851 J/m${}^{2}$ to 2059 J/m${}^{2}$ for CFRP. An SEM fractography revealed the presence of riverline and hackle patterns typical of mode I and mode II delamination, but also the presence of a highly damaged resin, giving a possible explanation for the high measured toughness. The Benzeggagh–Kenane and Power Law equations were used for curve fitting of the mixed-mode SERR data, allowing an estimation of the critical SERR envelope for any mode ratio. Both methods were found to give similar results.

The high measured toughness of the powder epoxy composites does not come with the disadvantage of increased resin cost, which is normally associated with the toughened epoxies found in the literature. These toughened epoxies can also require an additional processing step, which further adds to cost. Considering the processing advantages of the powder epoxy system, combined with a very high toughness, this study suggests that the material is a very good candidate resin system for structures for which delamination may be a concern.

## Figures and Tables

**Table 1:** Summary of tensile test results.

	GFRP			CFRP		
**Property**	**Result**	**Standard Deviation**	**COV**	**Result**	**Standard Deviation**	**COV**
Longitudinal Modulus E${}_{1}$ (GPa)	39.4	0.65	1.6%	123.0	6.7	5.4%
Longitudinal Strength $\sigma_{1}$ (MPa)	993	37	3.7%	1492	205	13.7%
Transverse Modulus E${}_{2}$ (GPa)	13.7	0.77	5.6%	8.47	0.37	4.4%
Transverse Strength $\sigma_{2}$ (MPa)	98.0	5.3	5.4%	31.5	1.9	6.0%
Shear Modulus G${}_{12}$ (GPa)	3.91	0.33	8.4%	4.30	0.67	15.6%
Shear Strength $\tau_{12}$ (MPa)	71.6	2.6	3.6%	64.2	1.2	1.9%
Poisson’s Ratio v${}_{12}$	0.29	0.093	32.1%	0.36	0.086	23.9%

**Table 2:** Summary of Compression Test Results.

	GFRP			CFRP		
**Property**	**Result**	**Standard Deviation**	**COV**	**Result**	**Standard Deviation**	**COV**
Longitudinal Modulus E${}_{1}$ (GPa)	37.8	4.2	11.1%	101.1	13.5	13.4%
Longitudinal Strength $\sigma_{1}$ (MPa)	518	53	10.2%	618	79	12.8%
Transverse Modulus E${}_{2}$ (GPa)	13.4	2.28	17.0%	6.73	1.96	29.1%
Transverse Strength $\sigma_{2}$ (MPa)	167	7.3	4.4%	130	5.9	4.5%

**Table 3:** Summary of DCB test results.

	Initiation						Propagation		
	**NL**			**5% Compliance**			**Mean R-Curve**		
	**Mean (J/m${}^{2}$)**	**STD (J/m${}^{2}$)**	**COV** **(%)**	**Mean (J/m${}^{2}$)**	**STD (J/m${}^{2}$)**	**COV** **(%)**	**Mean (J/m${}^{2}$)**	**STD (J/m${}^{2}$)**	**COV** **(%)**
GFRP	1377	103	7.5	1643	112	6.8	2048	268	13.1
CFRP	851	74	8.7	1070	142	13.3	1684	71	4.2

**Table 4:** Lever arm lengths for the MMB test setup.

	25% Mode II	50% Mode II	75% Mode II
Lever Length CFRP (mm)	94.2	50.8	35.3
Lever Length GFRP (mm)	96.3	51.4	35.5

**Table 5:** Summary of CFRP MMB test results.

	Initiation						Propagation		
	**NL**			**5% Compliance**			**Mean R-Curve**		
	**Mean** **(J/m${}^{2}$)**	**STD** **(J/m${}^{2}$)**	**COV** **(%)**	**Mean** **(J/m${}^{2}$)**	**STD** **(J/m${}^{2}$)**	**COV** **(%)**	**Mean** **(J/m${}^{2}$)**	**STD** **(J/m${}^{2}$)**	**COV** **(%)**
25% Mode II	1024	186	18.2	1529	192	12.5	2164	190	8.8
50% Mode II	1443	198	13.7	1976	150	7.6	2418	140	5.8
75% Mode II	2059	573	27.8	2562	187	7.3	3241	367	11.3

**Table 6:** Summary of GFRP MMB test results.

	Initiation						Propagation		
	**NL**			**5% Compliance**			**Mean R-Curve**		
	**Mean** **(J/m${}^{2}$)**	**STD** **(J/m${}^{2}$)**	**COV** **(%)**	**Mean** **(J/m${}^{2}$)**	**STD** **(J/m${}^{2}$)**	**COV** **(%)**	**Mean** **(J/m${}^{2}$)**	**STD** **(J/m${}^{2}$)**	**COV** **(%)**
25% Mode II	1499	125	8.4	1619	133	8.2	1792	186	10.4
50% Mode II	2132	143	6.7	2728	390	14.3	3374	427	12.7
75% Mode II	3118	215	6.9	4555	319	7.0	5151	404	7.8

**Table 7:** MMB test results for GF and CF epoxy composites.

	Material					
GFRP	UD GF/Epoxy with 5% 90° Fibres [32]	Mode Ratio	0%	28%	53%	72%
		Initiation SERR (J/m${}^{2}$)	118	340	580	1034
	UD GF/Epoxy hand layup [44]	Mode Ratio	0%	23%	47%	75%
		Initiation SERR (J/m${}^{2}$)	300	480	680	950
	UD GF/rubber toughened epoxy [45]	Mode Ratio	0%	27%	44%	
		Initiation SERR (J/m${}^{2}$)	440	473	743	
	UD GF/MWCNT toughened epoxy [46]	Mode Ratio	0%	33%	50%	67%
		Initiation SERR (J/m${}^{2}$)	235	666	719	1150
	UD GF/Powder Epoxy with 9.1% 90° Fibres and 2.9% Stitching	Mode Ratio	0%	25%	50%	75%
		Initiation SERR (J/m${}^{2}$)	1377	1499	2132	3118
CFRP	UD CF IM7/977-2 toughened epoxy [47]	Mode Ratio	0%	25%	50%	75%
		Initiation SERR (J/m${}^{2}$)	310	420	840	1280
	UD T700/SR8100 with 2.2% 90° Fibres [48]	Mode Ratio	0%	25%	50%	75%
		Initiation SERR (J/m${}^{2}$)	281	348	544	581
	UD T300/toughened epoxy prepreg (HS160) [49]	Mode Ratio	0%	28%	55%	85%
		Initiation SERR (J/m${}^{2}$)	250	350	510	710
	YTEC G40-800/5276-1 UD prepreg [50]	Mode Ratio	0%	25%	50%	75%
		Initiation SERR (J/m${}^{2}$)	320	404	721	1143
	UD CF/Powder Epoxy with 2.6% ±60° Fibres and 1.0% Stitching	Mode Ratio	0%	25%	50%	75%
		Initiation SERR (J/m${}^{2}$)	851	1024	1443	2059

## Data Availability

Data sharing is not applicable for this article.
